# Development of an SPRi Test for the Quantitative Detection of Cadherin 12 in Human Plasma and Peritoneal Fluid

**DOI:** 10.3390/ijms242316894

**Published:** 2023-11-29

**Authors:** Lukasz Oldak, Zenon Lukaszewski, Anna Leśniewska, Ksawery Goławski, Piotr Laudański, Ewa Gorodkiewicz

**Affiliations:** 1 Bioanalysis Laboratory, Faculty of Chemistry, University of Bialystok, Ciolkowskiego 1K, 15-245 Bialystok, Polandewka@uwb.edu.pl (E.G.); 2 Faculty of Chemical Technology, Poznan University of Technology, pl. Sklodowskiej-Curie 5, 60-965 Poznan, Poland; 3 1st Department of Obstetrics and Gynecology, Medical University of Warsaw, 02-091 Warsaw, Poland; 4 Department of Obstetrics, Gynecology and Gynecological Oncology, Medical University of Warsaw, 02-091 Warsaw, Poland; 5 OVIklinika Infertility Center, 01-377 Warsaw, Poland; 6 Women’s Health Research Institute, Calisia University, 62-800 Kalisz, Poland

**Keywords:** cadherin 12, SPRi biosensor, analytical test, biosensor development

## Abstract

A new method for the determination of cadherin 12 (CDH12)—an adhesive protein that has a significant impact on the development, growth, and movement of cancer cells—was developed and validated. The method is based on a biosensor using surface plasmon resonance imaging (SPRi) detection. A quartz crystal microbalance was used to analyze the characteristics of the formation of successive layers of the biosensor, from the linker monolayer to the final capture of CDH12 from solution. The association equilibrium constant (K_A_ = 1.66 × 10^11^ dm^3^ mol^−1^) and the dissociation equilibrium constant (K_D_ = 7.52 × 10^−12^ mol dm^−3^) of the anti-CDH12 antibody–CDH12 protein complex were determined. The determined analytical parameters, namely the values determining the accuracy, precision, and repeatability of the method, do not exceed the permissible 20% deviations specified by the aforementioned institutions. The proposed method is also selective with respect to possible potential interferents, occurring in up to 100-fold excess concentration relative to the CDH12 concentration. The determined Limit of Quantification (LOQ = 4.92 pg mL^−1^) indicates the possibility of performing quantitative analysis in human plasma or peritoneal fluid without the need to concentrate the samples; however, particular attention should be paid to their storage conditions, as the analyte does not exhibit high stability. The Passing–Bablok regression model revealed good agreement between the reference method and the SPRi biosensor, with ρ_Spearman_ values of 0.961 and 0.925.

## 1. Introduction

Cadherin 12 (CDH12) belongs to the cadherin family, which is a group of Ca^2+^-dependent homophilic adhesion receptors [1]. Cadherins regulate the turnover and reorganization of tissue structures. The structural integrity of solid tissues is maintained by interactions between cadherins and adjacent cells [2]. The cadherin superfamily contains several subgroups, of which classical cadherins are the most widely investigated. They include cadherins derived from different tissues, such as epithelial tissue (cadherin E), neural tissue (N-cadherin), and retinal tissue (cadherin R) [2].

CDH12 is a subtype of neural cadherin that is responsible for cell–cell contacts [3]. It has a molecular weight of 88 kDa and consists of 794 amino acid subunits [4]. CDH12 is an emerging potential biomarker that plays an essential role in tumorigenicity in colorectal cancer by promoting migration, invasion, adhesion, and angiogenesis [3,4]. It causes the progression of non-small-cell lung cancer [5] and salivary adenoid cystic carcinoma [6,7], as well as endometriosis and infertility [8]. CDH12 is also associated with some neuropsychiatric disorders [9,10]. All of this information has been obtained by the investigation of cancer tissue using immunochemistry [3,6] or semiquantitative Western blot [4]. The only circulating liquid so far investigated for CDH12 concentration was peritoneal liquid [8], where the marker was determined using an enzyme-linked immunosorbent assay kit (ELISA), showing a range between 2 and 10 ng mL^−1^. The authors of that study expressed the opinion that blood plasma is likely to be a better liquid medium for the determination of CDH12 as a biomarker. However, the marker has not yet been determined in human plasma or serum. Therefore, the development of new analytical tools suitable for the determination of CDH12, especially in blood plasma or serum, is highly desirable. 

The array surface plasmon resonance imaging (SPRi) technique, used with a suitable biosensor, appears to be a suitable tool for the determination of CDH12 in blood plasma or serum. SPR and SPRi biosensors enable the determination in blood plasma/serum of commonly used cancer biomarkers such as cancer antigen 125 (CA 125) [11], human epididymis protein 4 (HE 4) [12], and emerging cancer biomarkers such as aromatase [13], as well as numerous other biomarkers including cathepsins B, D, and G [14]. Other types of biosensors are also of great importance. They can be used to determine other important analytes, which may be, for example, neurotransmitters such as dopamine [15]. In contrast to ELISA, array SPRi is a label-free technique. Unlike the fluidic version of SPR, it enables the determination of these biomarkers within the necessary concentration ranges without signal enhancement or preliminary sample processing. An advantage of array SPRi is the linearity of the analytical signal and the ability to use very small samples of body fluid. The main difference between fluidic SPR and array SPRi is in the measuring process: in fluidic SPR a biosensor is created in situ and measuring is performed in the presence of processing solution, while in array SPRi the biosensor is created ex situ and the processing liquid is removed before measurement. This difference means that the array SPRi technique is more sensitive and enables the determination of a biomarker in body fluid without any sample processing or signal enhancement. Array SPRi uses chips that enable the measurement of multiple samples (usually 9 or 12) simultaneously. Each of these 9 or 12 measuring sites consists of 12 measuring points. A single result is an average for the 12 measuring points in the array, which significantly enhances the precision of that result.

Preliminary results show that a biosensor containing rat monoclonal anti-CDH12 antibody as a ligand, immobilized via cysteamine linker, responds well to CDH12 concentration and that the expected level of the marker in serum is of the order of ng mL^−1^.

CDH12 is identified as a classic type II cadherin due to the lack of His-Ala-Val at the N-terminus of the protein [16]. Type II cadherins belong to the family of cell–cell adhesion proteins. The subfamily of this type of cadherins is much less well known than the subfamily of type I cadherins. Structural studies of a large number of type II cadherins (including CDH8, CDH11, and CDH20) have shown that they form characteristic adhesive dimers. The only exception to this rule is VE-cadherin [17], which can also be determined with the SPR biosensor [18].

CDH12, like other cadherins, plays an important role in tissue homeostasis. It is responsible for cell adhesion and is necessary for cancer progression. CDH12 has also been shown to be associated with selected neuropsychiatric disorders [9]. 

Moreover, in the case of colorectal cancer, CDH12 affects the epithelial–mesenchymal transition (EMT), which is an important process in the pathogenesis of infertility and endometriosis [19,20,21]. 

There are no data regarding the exact mechanism of cleavage of CDH12 into extracellular and intracellular domains. However, by the example of another type II cadherin (E-cadherin) and bacterial infection, we elucidate the putative mechanism of CDH12 cleavage and the presence of free extracellular EC1–EC5 domains in body fluids. 

To attack the host organism, pathogenic bacteria must cross its barriers after binding to the epithelium. To do this, they must destroy the intercellular connections provided by cadherins, including E-cadherin. Bacteria use various mechanisms to dysregulate the homeostasis of cell–cell connections. In addition to bacteria, the cleavage of intercellular connections may occur after the secretion of metalloproteinases (e.g., ADAM-10) and proteases (e.g., HtrA), also present in inflammation and cancer, as a result of which the integrity of cell connections is weakened and body fluids with increasing amounts of extracellular cadherin domains enter [22,23].

Because CDH12 has been classified as a type II cadherin, we expect it to behave similarly to other proteins of this family and type and to retain its characteristic dimer-forming ability. For this reason, to carry out the experiments described below, we chose as a standard the CDH12 protein with a disulfide-linked homodimer structure and a monoclonal rat IgG_1_ antibody capable of binding CDH12 from its N-terminus.

The study of CDH12 concentration using the method proposed in this article and the previously published results on CDH12 content in the peritoneal fluid of women with endometriosis [8] are among the first to reveal the contents of CDH12 in human body fluids. Therefore, they may constitute a starting point for other researchers to become interested in CDH12, to broadly define and specify its function in various disease states, to determine its content in various biological materials, and to specify the predominant forms of CDH12 depending on the biological material and disease under study.

We describe here the development and subsequent stages of validation and verification of a new method for CDH12 determination in human body fluids. In view of its role in tumor life processes, migration, and angiogenesis as indicated above, CDH12 is a potential marker for the characterization of the patient’s condition, and CDH12 determinations have been carried out to date using several immunochemical methods (including ELISA) and semiquantitative Western blot.

Both ELISA and SPRi enable quantitative analysis of CDH12 content in human body fluids, but SPRi has several important advantages over ELISA. A comparative analysis was carried out on the basis of both of the aforementioned techniques for detecting IGF-1 in cow’s milk [24] and the method developed for CDH12 measurement. The method based on the SPRi biosensor requires small amounts of reagents and samples, and there is no need for special sample preparation. Only 3 µL of a sample is required for analysis (this may need to be diluted appropriately before testing). ELISA usually requires 100–200 µL. Labeled antibodies are not required for the SPRi biosensor. ELISA uses such antibodies, and the type of marker and reaction and incubation times are dictated by the selected detection method. Another advantage of the SPRi biosensor is the ability to regenerate its surface (for a certain number of cycles). This means that one plate can be used for completely different analyses or for a series of determinations of a single analyte in different samples. Moreover, ELISA is most often available in a version with 96 reaction microplates. This forces the analyst to collect research material for a certain period of time and store it in order to minimize the costs of analysis. The SPRi biosensor, owing to the possibility of easy and repeatable surface regeneration, enables the analysis of even just one sample, without wasting materials and reagents or generating costs of purchasing a new test for subsequent analyses.

## 2. Results and Discussion

### 2.1. Formation of Successive Biosensor Layers

**Figure 1:**
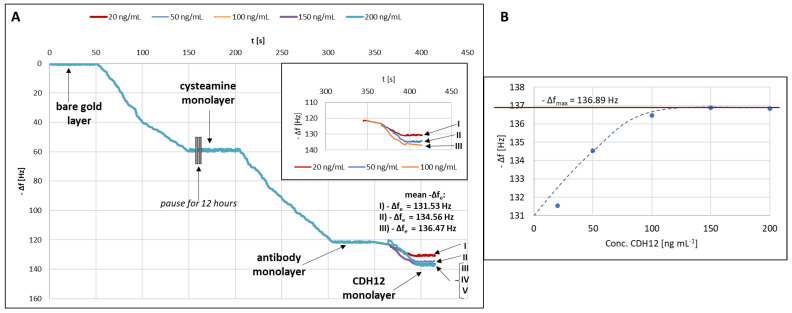
(**A**) Process of formation of successive biosensor layers. Curves I–V presents five different CDH12 concentrations; (**B**) Langmuir curve.

The process of formation of successive layers of the biosensor was monitored using quartz crystal microbalance (QCM). The measuring cell consists of two elements with two seals between them. The quartz crystal is placed in a designated place between these seals. Before measurements, both elements need to be tightened with three screws. After the measuring cell was assembled, 1 mL of deionized water was introduced into it, and the curve representing changes in the quartz resonator frequency (Δf) versus time was recorded until the signal stabilized. Signal stabilization was understood as a baseline drift of ±0.001 Hz. The stabilized value was taken as the pure gold baseline. Next, the water present in the cell was sucked out using a vacuum pump, and 1 mL of a 20 mM alcoholic solution of cysteamine hydrochloride was introduced (as in the SPRi studies). Δf stabilized again at a time of about 150 s, after which the recording of the measurement was stopped for 12 h. It is not possible for a cysteamine monolayer to be formed in only 100 s. In that time, probably all available particles reached the surface of the quartz resonator and settled on it, but did not bind to it in any way. The experiment was, therefore, stopped for 12 h to allow the cysteamine molecules to bind to the gold surface of the crystal, to enable the organization of the cysteamine monolayer, and to stabilize the entire system. Previous researchers have indicated that the time of monolayer formation varies depending on the length of the molecule chain and the conditions of the experiment. In general, the shorter the alkyl chain of a molecule, the faster and more readily it organizes on the surface (molecules of up to 12 carbon atoms within as little as an hour). However, to eliminate the influence of pressure and temperature (which were not controlled in detail in this study), and bearing in mind that the conditions described by other researchers apply to very dilute solutions, it was decided to leave the cysteamine solution in contact with Au(111) for at least 12 h [25,26,27,28]. After the indicated time, the excess cysteamine solution was gently removed and 1 mL of the antibody solution at a concentration of 30 ng mL^−1^ was introduced. Re-stabilization of Δf occurred after about 100 s. Again, the excess antibody solution was gently removed (with a pipette) and a 20 ng mL^−1^ solution of CDH12 was introduced. After each time that Δf stabilized, the excess solution was removed and the quartz crystal was gently rinsed with water. All operations were performed using an automatic pipette to maintain very mild conditions. The steps were repeated for a series of CDH12 solutions at concentrations of 20, 50, 100, 150, and 200 ng mL^−1^; however, for concentrations above 100 ng mL^−1^, no significant changes in Δf were observed. The graph of −Δf = f(t) showing the changes on the surface of the quartz resonator was plotted (Figure 1A), in addition to the relation −Δf = conc. CDH12, or the Langmuir curve, is needed to calculate the values of the equilibrium association constant (K_A_) and the equilibrium dissociation constant (K_D_) characterizing the tested antibody–CDH12 complex (Figure 1B).

Plotting the Langmuir curve makes it possible to determine the maximum frequency difference for the tested system under the adopted measurement conditions, which is necessary in order to determine K_A_. K_A_ was determined using the following Formula (1):

(1) $$K_{A}=\frac{∆f}{C\times ({∆f}_{max}-∆f)}$$

where:
K_A_ is the association equilibrium constant [dm^3^ mol^−1^];
Δf is the frequency difference between the ligand and CDH12 [Hz];
Δf_max_ is the maximum frequency difference between the ligand and CDH12 [Hz];
C is the CDH12 concentration [mol dm^−3^].
K_D_ was calculated using Equation (2):

(2) $$K_{D}=\frac{1}{K_{A}}$$

where:
K_D_ is the dissociation equilibrium constant [mol dm^−3^].

K_A_ and K_D_ values were calculated for three concentration levels (20, 50, and 100 ng mL^−1^), and their average value was then determined. The values of both constants were determined based on the calculation procedure described in [29].

The average values of K_A_ (1.66 × 10^11^ dm^3^ mol^−1^) and K_D_ (7.52 × 10^−12^ mol dm^−3^) were calculated, and show that the tested antibody–CDH12 complex is thermodynamically stable under the conditions of the tests. 

### 2.2. Investigation of Optimal Ligand Concentration

**Figure 2:**
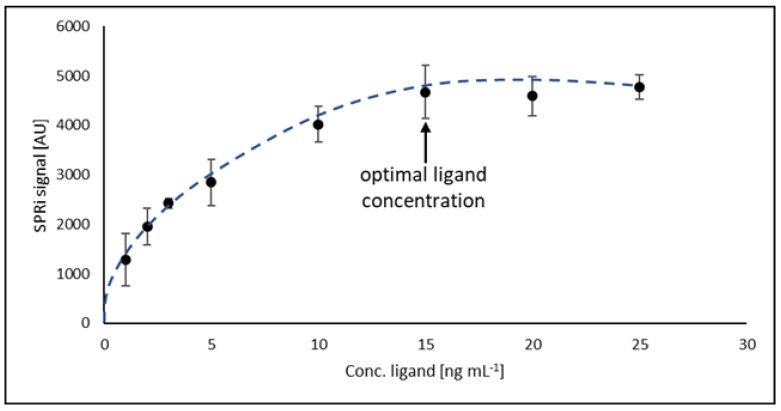
Optimal ligand concentration.

To determine the optimal concentration of the antibody (ligand) ensuring maximum surface saturation of the biosensor, the relationship between the detector response and the concentration of the antibody was examined. For this purpose, a series of antibody solutions with concentrations from 1.00 to 30.00 ng mL^−1^ was prepared. A drop (3 µL) of the antibody solution was applied to the active sites of the biosensor and incubated as previously described. Then, 3 µL of CDH12 solution with a concentration of 2 ng mL^−1^ was applied according to the previously described procedure. All solutions were tested at pH = 7.40 and at room temperature. It was determined that the optimal value of antibody concentration is 15 ng mL^−1^, and above this value, there is no covalent binding of antibodies to the biosensor surface. Figure 2 shows the investigation of optimal ligand concentration.

### 2.3. Method Calibration, Limit of Detection (LOD), and Limit of Quantification (LOQ)

**Figure 3:**
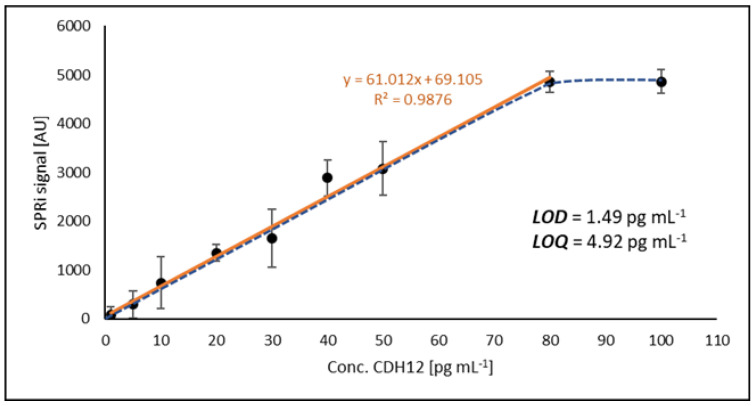
Calibration curve and LOD and LOQ values (solid orange line presents analytically useful range of the calibration curve).

For calibration of the method, a series of standard solutions in the concentration range 1–100 pg mL^−1^ was prepared. Above a concentration of 80 pg mL^−1^ a plateau was observed; hence, the rectilinear range lies between 1 and 80 pg mL^−1^. Figure 3 shows the rectilinear range of the obtained calibration curve along with the LOD and LOQ values. The error bars are standard deviations from successive readings of SPRi signals.

The LOD and LOQ values were determined by calculating the concentration values that the device’s detector would read based on the signal generated by the blank sample (PBS buffer). Five replicates of the blank were performed and then the SD (standard deviation) of the concentration measurements was determined. LOD and LOQ were determined using the following relationships: LOD = 3 × SD and LOQ = 3 × LOD. The calculated limits determine the useful range of the developed analytical method, which is from 4.92 to 80 pg mL^−1^.

### 2.4. Accuracy and Precision

Precision and intra-run accuracy were determined by quantifying four concentration levels within the analytically useful range of the calibration curve (5.00, 10.00, 40.00, and 80.00 pg mL^−1^). Therefore, we prepared a series of standard solutions with the aforementioned concentrations in PBS buffer. Determinations of intra-series accuracy and precision were performed on the same day, immediately after preparing the solutions. The solutions were then stored at 4 °C until being assayed for inter-run accuracy and precision. The determinations for each concentration level were repeated three times. For inter-run precision and accuracy, analogous determinations were carried out for 2 consecutive days. The measure of precision is the coefficient of variation (CV) calculated from Equation (3). The measure of accuracy is the relative percentage error (δ) determined from Equation (4), as recommended by the guidelines for the validation of bioanalytical methods issued by the Food and Drug Administration (FDA) and the European Medicines Agency (EMA). These guidelines also contain detailed data on the acceptance criteria for the results [30,31]. The data obtained in the analyses are presented in Table 1.

(3) $$CV=\frac{SD}{C_{mean}}\times 100\%$$

(4) $$\delta =\frac{\left|C_{mean}-C_{real}\right|}{C_{real}}\times 100\%$$

where:
CV is the coefficient of variation;
SD is the standard deviation;
δ is the relative percentage error;
C_mean_ is the mean concentration;
C_real_ is the real concentration.

The precision values do not exceed ± 20% CV, and the accuracy values do not exceed ±20% of the nominal (assumed) concentration. Based on these data, it was concluded that the developed method is precise and accurate.

### 2.5. Repeatability

The repeatability of the developed method was determined by measuring the concentration of each of the four selected natural samples three times. Two of the samples were plasma and two were peritoneal fluid. Each sample was diluted 1000 times. Each repetition of the measurement was performed on the same biosensor plate but after regeneration of the sites where the reactions took place. Regeneration involves destroying the amide bonds between cysteamine and the antibody. Before proceeding with further repetitions of the determinations, the procedure of coating the biosensor with an antibody had to be carried out. The results of the determinations are presented in Table 2.

According to FDA and EMA guidelines, the value determining the repeatability of a method is the coefficient of variation (CV). The acceptance criteria issued by both institutions assume a maximum CV of 20% for immunoassay methods. The method developed by our team is characterized by a CV (for repeatability) of 4.81% to 5.92% (four independent samples were tested, with each measurement repeated three times). The presented values show, in accordance with the FDA and EMA guidelines, that the method developed for the determination of CDH12 gives reproducible values of the tested analyte concentration, both in human plasma and in human peritoneal fluid.

### 2.6. Analyte Stability

We tested the stability of the analyte by exposing a natural sample to harmful factors selected as being most likely to occur in an analytical laboratory. These factors included: leaving the sample for 2 and 24 h at room temperature, changing the pH of the sample from 7.40 to 5.00 and 9.50, and a freezing and thawing cycle repeated five times, with the sample brought to room temperature each time. Since the influence of four different harmful factors was to be determined, the primary test sample was divided into four equal parts. The initial average concentration of CDH12 in the sample prior to exposure was 20.62 ng mL^−1^. Each measurement was repeated three times, the average concentration was calculated, and Δ% was determined in accordance with Formula (5) and the FDA and EMA recommendations.

(5) $$\Delta \%=\frac{C_{\mathrm{initial}\mathrm{concentration}}-C_{\mathrm{harmful}\mathrm{conditions}}}{C_{\mathrm{initial}\mathrm{concentration}}+C_{\mathrm{harmful}\mathrm{conditions}}}\times 200$$

The smallest error (1.71%) was generated by leaving the sample for 2 h at room temperature. A noticeably greater change (14.46%) in the result for CDH12 concentration was obtained by leaving the sample for 24 h at a temperature of about 23 °C. Changing the pH value from neutral to 5.00 and 9.50 generated errors in the determinations of 41.63% and 43.50%, respectively. However, the greatest impact on the result for CDH12 content in the natural sample came from exposure to thawing and freezing cycles, in which case an error as high as 53.29% was recorded in the determinations made using the developed method.

### 2.7. Selectivity

The antibody manufacturer has determined that its product does not cross-react with other CDH family proteins. However, we additionally examined the effect of two other proteins, neuropilin 1 (NRP-1) and vascular endothelial growth factor (VEGF-A), affecting the metastasis of cancer and angiogenesis. There is very little known about the role of CDH12 in the angiogenesis process, but a connection with the angiogenesis of other cadherins has been proven. We, therefore, selected VEGF-A and NRP-1 as interferents because we assume that CDH12 may perform similar functions in the processes of angiogenesis and metastasis as other cadherins [3,32,33,34]. We also tested the effect of human albumin, a protein that accounts for over 50% of all proteins in the human body. 

The physiological concentration of VEGF-A is 62–707 pg mL^−1^ in serum or 0–115 pg mL^−1^ in plasma [35], while the physiological average concentration of NRP-1 in plasma is approximately 150 ng mL^−1^ [36]. VEGF-A concentrations, as they are an order of magnitude lower than the CDH12 concentrations determined using the developed method, should not falsify the results. However, NRP-1 concentrations significantly exceed CDH12 levels; therefore, the effect of excess concentration of this potential interferent was specifically investigated.

A series of standard solutions were prepared, each containing CDH12 and a selected potential interferent in a concentration ratio of 1:1, 1:10, or 1:100. The prepared mixtures were left for 24 h in a refrigerator (at 4 °C) to allow the new system to stabilize. Each measurement was repeated three times, and the average concentration and recovery values were calculated using Equation (6). The data set obtained is summarized in Table 3.

(6) $$recovery=\frac{C_{\mathrm{mean}\mathrm{CDH}12}}{C_{\mathrm{theoretical}\mathrm{CDH}12}}\times 100\%$$

The average recovery values are in the range of 98.36–102.26%. This shows that excess amounts of the potential interferents relative to the concentration of CDH12 have little influence on the final result of the assays. The developed method is, therefore, resistant to interference caused by the presence of other proteins up to a ratio of 1:100 (CDH12: interferent; Conc./Conc.).

### 2.8. CDH12 Quantification and Comparison of Methods

The matrix effect was tested on 10 plasma samples and 10 peritoneal fluid samples (both patient samples and control samples). The role of the substitute matrix was played by PBS, in which CDH12 solutions were prepared with concentrations previously determined in plasma and peritoneal fluid. Then, a known amount of CDH12 was added to each sample (in the real and substitute matrices) and the determinations were carried out again, the results of which are presented in Table 4.

The differences between recoveries from real matrix samples and substitute matrix samples are small and do not exceed 10%; hence, we conclude that the sample matrix does not influence the determination result. 

### 2.9. CDH12 Quantification and Comparison of Methods

The newly developed method was used to determine CDH12 on a population of 30 samples, consisting of 15 plasma samples and 15 samples of peritoneal fluid. Plasma and peritoneal fluid samples (patient and control samples) were from the same patients. Samples were prepared for analysis in accordance with the procedure previously described.

Similarly, determinations of the same samples were made using an available comparative method, ELISA. The assays were carried out in accordance with the instructions provided by the manufacturer.

**Figure 4:**
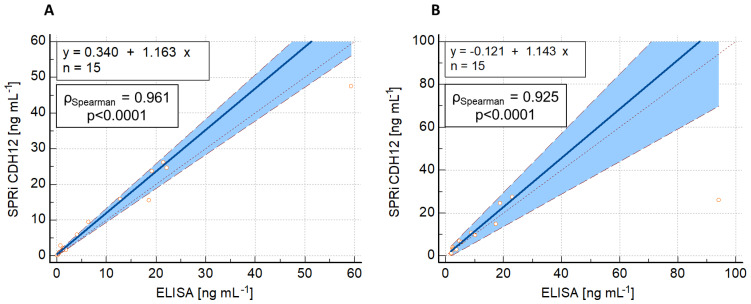
Passing–Bablok regression curves comparing the results of the SPRi CDH12 and ELISA methods performed on (**A**) plasma samples and (**B**) peritoneal fluid samples. The blue solid line marks the Passing–Bablok regression curve; the black fine dashed line indicates where the bias would be zero (SPRi CDH12 = ELISA).

To assess the compatibility of the results from both methods, a Passing–Bablok regression analysis was performed, with SPRi CDH12 as the candidate method (y) and ELISA as the reference method (x). The results of the regression are shown in Figure 4.

The CDH12 concentration in each of the 15 plasma samples and 15 peritoneal fluid samples was determined twice with the SPRi CDH12 assay, and the average concentration value was taken. ELISA uses spectrophotometry as a detection method and, in addition to the antibody capturing CDH12 from the solution, it requires a second, labeled antibody for further reactions with the label to lead to a color reaction. A significant deviation in one result may, therefore, be caused by the hook effect, i.e., an excess of labeled antibodies and their non-specific binding to the capture antibodies or the walls of the reaction well, which was poorly washed. These antibodies still have the ability to participate in color reactions even though they did not bind to CDH12 to form a sandwich complex.

The above data show that the deviation of the concentrations obtained is not constant. It increases proportionally with increasing concentration, and this is particularly visible for determinations in peritoneal fluid. This may be due to the presence of one clear outlier for each method—at a concentration of 94.13 ng mL^−1^ for ELISA and 25.96 ng mL^−1^ for SPRi CDH12—although the Passing–Bablok regression model is resistant to outliers. In both cases, however, very good agreement between the methods was observed (ρ_Spearman_ = 0.961 and 0.925).

The table below (Table 5) characterizes the new CDH12 assay method in accordance with WHO’s ASSURED criteria [37,38].

### 2.10. Biosensor Regeneration

**Figure 5:**
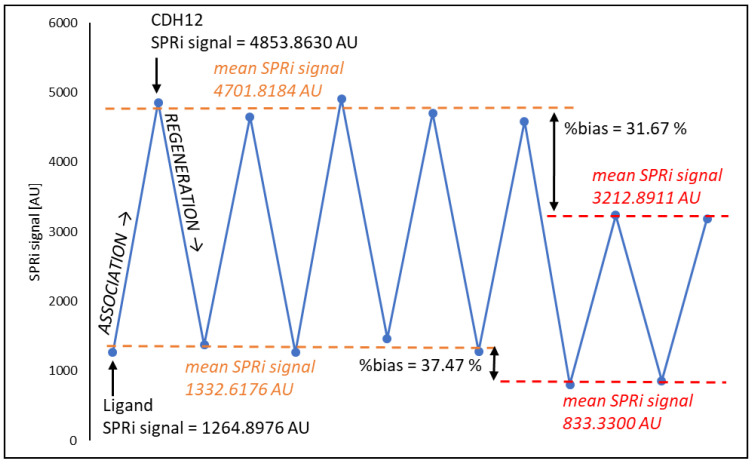
Regeneration efficiency.

In the first stage of regeneration, the biosensor plate was removed from the glass prism and very thoroughly cleaned to remove residues of immersion oil. This step was carried out using 96% ethyl alcohol. The reaction sites were then regenerated using 50 mM NaOH with 0.05% Tween-20. The solution was heated to 60 °C and the plates were immersed in it for 5 min. After this time, the plates were rinsed in distilled water (T = 60 °C) for 5 min, in ethyl alcohol (96%, T = 23 °C) for 30 s, and again in heated distilled water (t = 5 min, T = 60 °C). Finally, the plates were dried in a stream of argon. The regeneration efficiency was checked and the number of possible regeneration cycles was determined. For this purpose, the ability to return to the base signal generated by the presence of the antibody on the biosensor surface and the ability to return to the signal generated by CDH12 at a concentration of 2 ng mL^−1^ (standard solution) were tested, with differences up to a maximum of 10% allowed relative to the average value of the results against significant changes in the SPRi signal. The results are shown in Figure 5. The approach to regenerating the sensor surface using NaOH has also been used by another research group [39], which also reported high regeneration efficiency and the possibility of repeated use of the biosensor. 

After five regeneration cycles, a clear decrease in the SPRi signal for the ligand layer was observed, which resulted in a decrease in the number of CDH12 molecules captured from the solution. The linker (cysteamine) monolayer was probably significantly disorganized and destroyed. This results in fewer ligands on the biosensor surface and, therefore, fewer CDH12 molecules bound to them.

## 3. Materials and Methods

### 3.1. Chemical Reagents and ELISA

The basis of the developed biosensor was a 1 mm thick glass plate with a 50 nm sputtered gold layer supplied by Ssens (Enschede, The Netherlands). Monoclonal recombinant rat anti-CDH12 antibody (Cat. No. MAB2240) and recombinant human CDH12 protein (Cat. No. 2240-CA), each >95% pure, were purchased from R&D Systems. Other reagents, including cysteamine hydrochloride, 1-ethyl-3-(3-dimethylaminopropyl)carbodiimide (EDC), *N*-hydroxysuccinimide (NHS), bovine serum albumin (BSA), 0.2 M carbonate buffer with pH = 8.50, and Tween-20 were obtained from Merck (Darmstadt, Germany). Absolute ethyl alcohol (99.8%) and 96% ethyl alcohol were purchased from Pol Aura (Morag, Poland), and buffered saline solution (PBS) with pH = 7.40 was purchased from Biomed (Lublin, Poland). Immersion oil 518 Immersol was obtained from Zeiss (Jena, Germany). Each of the reagents had a purity above 98%. Deionized water was obtained from an HLP Smart system (Straszyn, Poland). The ELISA kit was obtained from SunRedBio (Shanghai, China; analytical parameters: LOD = 0.05 ng mL^−1^, CV_intra-run_ < 10%, CV_inter-run_ < 12%, assay range 0.08–20.00 ng mL^−1^); absorbance readings (λ = 450 nm) were made with a MULTISCAN GO microplate reader (Thermo Fisher Scientific, Vantaa, Finland).

### 3.2. SPRi Instrument

The SPRi analyzer is an optical device in which the radiation source is a laser diode with a wavelength of λ = 635 nm and a maximum power of 8 mW, which is controlled by an appropriate controller with temperature control. The radiation then falls on the collimator (λ = 635 nm, f = 35.41 mm) and the radiation beam expander (400–650 nm). Then, after passing through a polarizer, which allows the separation of the radiation component responsible for p-polarization, the light is reflected from the biosensor surface. The reflected beam goes to a bi-telecentric lens and then directly to the detector—a monochrome CCD camera with a resolution of 1.4 MP. The device was constructed by our research team. The data acquisition time was 3 min. This is the time needed to remove excess liquid from the surface of the active sites of the biosensor, properly rinse these sites, and save the image of the active sites. To maintain the high sensitivity of the method, data acquisition should be performed before the expiry of the time, which guarantees the stability of the analyte.

### 3.3. Software

The biosensor images used to determine the final analytical signal were acquired from a CCD camera using the software provided by the camera’s manufacturer, Basler Beteiligungs-Gmbh & Co., KG (Ahrensburg, Germany). The free software ImageJ v.1.51k (NIH, USA) was used for the mathematical processing of the obtained images. The operation of a quartz microbalance (QCM) was supported by NOVA 2.1.2 software from Metrohm Autolab B.V. (Utrecht, The Netherlands). Mathematical operations were performed using MS Excel (Redmond, WA, USA), and statistical analysis was carried out using MedCalc^®^ Statistical Software version 22.009 (MedCalc Software Ltd., Ostend, Belgium, version 22.009). 

### 3.4. Biological Material 

Plasma and peritoneal fluid were taken from patients of the Angelius Provita Hospital in Katowice (Poland). The patients had undergone laparoscopy and been diagnosed with endometriosis according to the current classification, which was finally confirmed by histopathological examination. The control samples were plasma and peritoneal fluid from patients without endometriosis but with infertility, pelvic pain, or ovarian cyst. Appropriate consent was obtained from the Ethics Committee of the Medical University of Warsaw (AKBE/133/2020) to conduct research using this biological material. 

### 3.5. Methods

The process of formation of successive layers of the biosensor was investigated using a quartz crystal microbalance (QCM), as described in more detail in the next section.

Interactions between the anti-CDH12 monoclonal antibody and CDH12 were recorded using a spectrometer that induces the effect of surface plasmon resonance. Each subsequent molecule bound to the biosensor surface causes changes in the intensity of plasmon oscillations, which results in changes in the refractive index. The equipment used records images of the biosensor, in which the elements of interest are the so-called active sites, that is, places where desired chemical reactions take place. Changes in the refractive index are represented in the case of our apparatus by changes in the brightness intensity of the aforementioned active sites. Thus, the final analytical signal is the difference in the intensity of a given active site after and before the analyte–antibody binding reaction. The biosensor used in the research had 12 such active sites, which enabled the determination of 12 samples at the same time. Moreover, in accordance with the assumptions of the SPR imaging technique, photos of the biosensors were recorded at one, previously experimentally determined, optimal SPR angle, that is, the angle at which the signal difference between the ligand–analyte immune complex and the ligand itself was the highest.

The base of the biosensor, a glass plate with a layer of gold, required prior cleaning and printing of a light-hardening polymer (Elpemer SD 2057), which enabled the determination of 12 measurement locations on the biosensor. The plate was then appropriately dried (at 65 °C for a time of 1 h) and was irradiated with UV rays for 5 min. In the last step, the plate was rinsed with absolute ethanol and deionized water and dried in a stream of argon with purity >99.98%. The plate prepared in this way is suitable for the further stages of the research. 

The next process is the immobilization of a self-assembled linker monolayer (cysteamine) on the surface of the biosensor. For this purpose, a 20 mM alcoholic solution of cysteamine hydrochloride was prepared, in which a plate with separate measurement points was immersed in a sealed vessel. This was left for at least 12 h at a temperature of approximately 23 °C (laboratory temperature). The next day, the plate was carefully and gently rinsed in absolute ethanol and deionized water and dried in a stream of argon. 

The cysteamine monolayer located on the surface enables the covalent bonding of the biorecognition element—the monoclonal antibody—due to the reaction of the amino group of the cysteamine (×NH_2_) with the carboxyl group at the end of the antibody’s heavy chain (−COOH). As a result of this reaction, a peptide bond (−CONH) is formed. To enable this reaction, the carboxyl group of the antibody was modified with a mixture of 0.4 M EDC/0.4 M NHS in a volume ratio of 1:1, which was added to the prepared antibody in 0.2 M carbonate buffer solution. This step led to the formation of semi-stable N-succinimidyl esters. Then, 3 µL of the modified antibody solution was applied to the active sites of the biosensor. The whole was incubated for 1 h at 37 °C. After this process, the active sites of the biosensor were washed five times with deionized water, a 1 mM BSA solution was applied to eliminate non-specific adsorption, and the active sites of the biosensor were rinsed again with deionized water. Semi-stable N-succinimidyl esters react easily and quickly with amino groups (present on the surface of the biosensor due to cysteamine immobilization), resulting in the formation of the aforementioned peptide bond. 

Once the antibody is bound to the self-assembled cysteamine monolayer, the biosensor is useful for quantification. The interaction between the antibody (ligand) and CDH12 proceeds by binding the epitope (present in the analyte, CDH12) by the paratope (present in the ligand). The biosensor images were recorded according to the following scheme: (i) placing a drop of immersion oil (n_D_ = 1.518) on a glass prism (n_D_ = 1.510) located in the SPRi spectrometer—to prevent the formation of a glass-to-air interface; (ii) placing the biosensor on a prism, locking it in one position and recording images when there is a covalently bound ligand on the surface of the biosensor; (iii) applying 3 µL of the tested samples to the active sites of the biosensor and leaving them for 5 min; (iv) gently removing excess fluid and gently rinsing each area once with a 3 µL drop of deionized water; and (v) registration of biosensor images and mathematical processing, according to an algorithm based on subtraction of the analytical signal read for the site with bound CDH12 from the signal for the analogous site with only the ligand present.

The procedure for determining CDH12 using the developed method in human plasma and human peritoneal fluid required prior 1000-fold dilution of the samples with the neutral buffer PBS. The multiplicity of dilution was selected experimentally, and it was in these conditions that the repeatability of the method and the stability of the analyte were determined. Assuming that we use a biosensor with a previously prepared cysteamine monolayer, the analytical response time of the biosensor is 1 h and 10 min. This is the time necessary to bind the antibody to the biosensor surface, bind the analyte, perform the planned washing cycles, and acquire data. 

The designed biosensor was used many times, after prior regeneration, which focused on the basic hydrolysis of the peptide bond formed between the linker and the ligand. Regeneration was carried out by immersing the biosensor plate in a 50 mM NaOH aqueous solution with the addition of 0.05% Tween-20. The solution was heated to 60 °C, and the biosensor plate (previously thoroughly cleaned of immersion oil) was immersed in it for 5 min. The plate was then carefully rinsed with deionized water and absolute ethyl alcohol and dried in a stream of argon.

**Figure 6:**
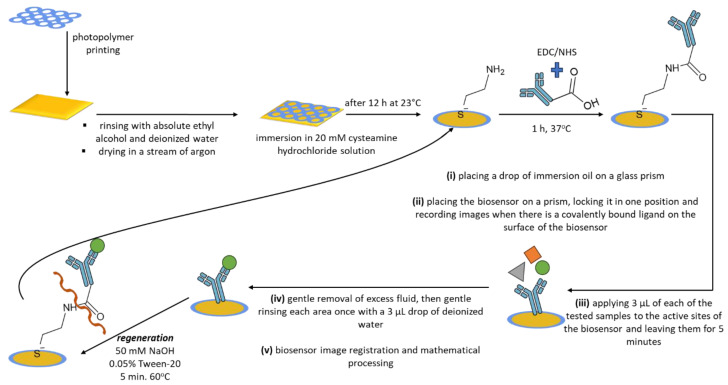
Schematic representation of the procedures for biosensor preparation, CDH12 quantitative analysis, and biosensor regeneration.

All stages of the procedure described above are shown in Figure 6.

## 4. Conclusions

A new, rapid, label-free method for the determination of CDH12 in human plasma and peritoneal fluid was developed based on an immuno-reaction between an anti-CDH12 monoclonal antibody and human CDH12. Our team conducted a comprehensive validation of the developed method. The work began with the characterization of the build-up of successive layers of the biosensor and the determination of the basic thermodynamic parameters of the emerging antibody–CDH12 system. QCM was used for these experiments. The curves showing changes in the frequency of the quartz resonator over time confirm the build-up of successive layers of the biosensor, while the values of K_A_ = 1.66 × 10^11^ dm^3^ mol^−1^ and K_D_ = 7.52 × 10^−12^ mol dm^−3^ prove the thermodynamic stability of the complex. Using the proposed method, it is possible to determine CDH12 even in samples with trace amounts of that protein (LOQ = 4.92 pg mL^−1^), and because the analytically useful range runs from LOQ to 80 pg mL^−1^, determinations in materials with higher concentrations of CDH12 require the prior dilution of samples with PBS buffer at pH = 7.40. The quantities characterizing the precision (CV) and accuracy (δ) clearly prove the high precision and accuracy of the method: the maximum value of CV is 9.13% and that of δ is 4.11%. Both of these values are not even close to the limit value (20%) laid down in the FDA and EMA guidelines. The CV value characterizing the repeatability of the method is within the range of 4.81–5.92%, while the FDA and EMA guidelines allow CV ≤ 20%; this indicates the good repeatability of the SPRi CDH12 method. To ensure maximum reliability of the obtained CDH12 concentration results, we examined the impact of potentially harmful conditions and laboratory errors on the final result of the analysis performed using the developed method. The result of the quantitative analysis of CDH12 using the developed method is mainly influenced by the change in pH value and cyclic freezing and thawing of the sample. Moreover, the method is selective and the CDH12 concentration results are comparable to the reference method. 

## Figures and Tables

**Table 1:** Intra-run and inter-run precision and accuracy.

Intra-Run Precision and Accuracy				
CDH12 concentration (pg mL^−1^)				
	5	10	40	80
Precision (CV) (%)	9.13	4.91	3.82	2.08
Accuracy (δ) (%)	1.44	4.11	3.29	3.09
Inter-run precision and accuracy—day 1				
Precision (CV) (%)	8.66	4.93	3.61	2.07
Accuracy (δ) (%)	3.89	3.67	2.54	2.72
Inter-run precision and accuracy—day 2				
Precision (CV) (%)	8.71	4.95	3.74	2.04
Accuracy (δ) (%)	3.36	3.37	1.10	1.29
Averaged values				
Precision (CV) (%)	8.83	4.93	3.72	2.06
Accuracy (δ) (%)	2.90	3.72	2.31	2.36

**Table 2:** Analytical parameters characterizing the repeatability of the method.

	Plasma		Peritoneal Fluid	
	Sample 1	Sample 2	Sample 3	Sample 4
Repeat 1	21.72	12.83	6.28	20.49
Repeat 2	19.77	13.02	5.59	21.85
Repeat 3	20.36	11.89	5.94	19.42
Mean Concentration (ng mL^−1^)	20.62	12.58	5.94	20.59
SD (ng mL^−1^)	1.00	0.61	0.35	1.22
CV (%)	4.85	4.81	5.81	5.92

**Table 3:** Influence of potential interferents on CDH12 assays.

CDH12: Interferent	Concentration Ratio	C_theoretical_ CDH12 (pg mL^−1^)	C_mean_ CDH12 (pg mL^−1^)	Recovery (%)	Mean Recovery (%)
CDH12: VEGF-A	1:1	30.00	28.76	95.87	98.36
	1:10		32.64	108.80	
	1:100		27.12	90.40	
CDH12: NRP-1	1:1		31.22	104.07	102.26
	1:10		27.75	92.50	
	1:100		33.06	110.20	
CDH12: human albumin	1:1		29.18	97.27	101.81
	1:10		30.91	103.03	
	1:100		31.54	105.13	

**Table 4:** The influence of matrix on determinations.

Plasma							
Sample Number	C_unenriched_ (ng mL^−1^)	C_added_ (ng mL^−1^)	Real Matrix		Substitute Matrix		Recovery Difference (%)
			C_enriched_ (ng mL^−1^)	Recovery (%)	C_enriched_ (ng mL^−1^)	Recovery (%)	
1	21.72	20	42.45	103.65	42.18	102.3	1.35
2	6.44		27.14	103.5	26.89	102.25	1.25
3	20.2		41.08	104.4	40.34	100.7	3.7
4	24.65		44.94	101.45	45.37	103.6	2.15
5	17.54		37.12	97.9	38.02	102.4	4.5
6	12.83		33.51	103.4	33.43	103	0.4
7	1.53		22.71	105.9	21.93	102	3.9
8	4.87		25.07	101	25.11	101.2	0.2
9	1.65		21.94	101.45	21.49	99.2	2.25
10	2.07		22.61	102.7	21.83	98.8	3.9
**Peritoneal Fluid**							
**Sample Number**	**C_unenriched_ (ng mL^−1^)**	**C_added_** **(ng mL^−1^)**	**Real Matrix**		**Substitute Matrix**		**Recovery Difference (%)**
			**C_enriched_** **(ng mL^−1^)**	**Recovery (%)**	**C_enriched_** **(ng mL^−1^)**	**Recovery (%)**	
1	8.61	20	29.37	103.8	29.02	102.05	1.75
2	24.36		45.05	103.45	44.97	103.05	0.4
3	16.76		37.21	102.25	36.89	100.65	1.6
4	20.49		40.92	102.15	40.16	98.35	3.8
5	25.96		46.7	103.7	46.27	101.55	2.15
6	9.59		31.33	108.7	30.64	105.25	3.45
7	2.89		23.4	102.55	23.06	100.85	1.7
8	2.99		23.67	103.4	23.26	101.35	2.05
9	2.38		23.19	104.05	22.84	102.3	1.75
10	4.81		24.99	100.9	24.37	97.8	3.1

**Table 5:** WHO’s ASSURED criteria for SPRi CDH12 tests.

Test Parameters	SPRi CDH12 Plasma	SPRi CDH12 Peritoneal Fluid
Diagnostic target	Antigen	Antigen
Test format	SPRi 2D array biosensor	SPRi 2D array biosensor
Affordable	Cost less than the ELISA kit	Cost less than the ELISA kit
Sensitive (%) ^a^	77.8	66.7
Specific (%) ^a^	83.3	100
User-friendly	Yes, 4 steps	Yes, 4 steps
Rapid and robust (min)	1 h and 10 min	1 h and 10 min
Equipment free	No	No
Deliverable	Used in labs	Used in labs

^a^ ROC analysis.

## Data Availability

Data are contained within the article.

## References

[1] Gumbiner B.M. (1996). Cell Adhesion: The Molecular Basis of Tissue Architecture and Morphogenesis. Cell.

[2] Leckband D., Prakasam A. (2006). Mechanism and Dynamics of Cadherin Adhesion. Annu. Rev. Biomed. Eng..

[3] Zhao J., Li P., Feng H., Wang P., Zong Y., Ma J., Zhang Z., Chen X., Zheng M., Zhu Z. (2013). Cadherin-12 Contributes to Tumorigenicity in Colorectal Cancer by Promoting Migration, Invasion, Adhersion and Angiogenesis. J. Transl. Med..

[4] Ma J., Zhao J., Lu J., Wang P., Feng H., Zong Y., Ou B., Zheng M., Lu A. (2016). Cadherin-12 Enhances Proliferation in Colorectal Cancer Cells and Increases Progression by Promoting EMT. Tumor Biol..

[5] Bankovic J., Stojsic J., Jovanovic D., Andjelkovic T., Milinkovic V., Ruzdijic S., Tanic N. (2010). Identification of Genes Associated with Non-Small-Cell Lung Cancer Promotion and Progression. Lung Cancer.

[6] Wang J.F., She L., Su B.H., Ding L.C., Zheng F.F., Zheng D.L., Lu Y.G. (2011). CDH12 Promotes the Invasion of Salivary Adenoid Cystic Carcinoma. Oncol. Rep..

[7] Guo B., Qi M., Huang S., Zhuo R., Zhang W., Zhang Y., Xu M., Liu M., Guan T., Liu Y. (2021). Cadherin-12 Regulates Neurite Outgrowth Through the PKA/Rac1/Cdc42 Pathway in Cortical Neurons. Front. Cell Dev. Biol..

[8] Goławski K., Soczewica R., Kacperczyk-Bartnik J., Mańka G., Kiecka M., Lipa M., Warzecha D., Spaczyński R., Piekarski P., Banaszewska B. (2022). The Role of Cadherin 12 (CDH12) in the Peritoneal Fluid among Patients with Endometriosis and Endometriosis-Related Infertility. Int. J. Env. Res. Pub. Health.

[9] Thalmeier A., Dickmann M., Giegling I., Schneider B., Hartmann A.M., Maurer K., Schnabel A., Kauert G., Möller H.J., Rujescu D. (2008). Gene Expression Profiling of Post-Mortem Orbitofrontal Cortex in Violent Suicide Victims. Int. J. Neuropsychopharmacol..

[10] Lydall G.J., Bass N.J., McQuillin A., Lawrence J., Anjorin A., Kandaswamy R., Pereira A., Guerrini I., Curtis D., Vine A.E. (2011). Confirmation of Prior Evidence of Genetic Susceptibility to Alcoholism in a Genome-Wide Association Study of Comorbid Alcoholism and Bipolar Disorder. Psychiatr. Genet..

[11] Diken Gür S., Bakhshpour M., Denizli A. (2022). Nanoscale SPR Sensor for the Ultrasensitive Detection of the Ovarian Cancer Marker Carbohydrate Antigen 125. New J. Chem..

[12] Duan R., Xi M. (2020). A Novel Label-Free Biosensor for Detection of HE4 in Urine Based on Localized Surface Plasmon Resonance and Protein G Directional Fixed. J. Nanomater..

[13] Gorodkiewicz E., Sankiewicz A., Laudański P. (2014). Surface Plasmon Resonance Imaging Biosensors for Aromatase Based on a Potent Inhibitor and a Specific Antibody: Sensor Development and Application for Biological Material. Cent. Eur. J. Chem..

[14] Laudanski P., Gorodkiewicz E., Ramotowska B., Charkiewicz R., Kuzmicki M., Szamatowicz J. (2013). Determination of Cathepsins B, D and G Concentration in Eutopic Proliferative Endometrium of Women with Endometriosis by the Surface Plasmon Resonance Imaging (SPRI) Technique. Eur. J. Obstet. Gyn. Reprod. Biol..

[15] Ravariu C. (2023). From Enzymatic Dopamine Biosensors to OECT Biosensors of Dopamine. Biosensors.

[16] Gessner R., Tauber R. (2000). Intestinal Cell Adhesion Molecules. Liver-Intestine Cadherin. Ann. N. Y. Acad. Sci..

[17] Brasch J., Katsamba P.S., Harrison O.J., Ahlsén G., Troyanovsky R.B., Indra I., Kaczynska A., Kaeser B., Troyanovsky S., Honig B. (2018). Homophilic and Heterophilic Interactions of Type II Cadherins Identify Specificity Groups Underlying Cell-Adhesive Behavior. Cell. Rep..

[18] Fathi F., Rezabakhsh A., Rahbarghazi R., Rashidi M.R. (2017). Early-Stage Detection of VE-Cadherin During Endothelial Differentiation of Human Mesenchymal Stem Cells Using SPR Biosensor. Biosens. Bioelectron..

[19] Matsuzaki S., Darcha C. (2012). Epithelial to Mesenchymal Transition-like and Mesenchymal to Epithelial Transition-like Processes Might be Involved in the Pathogenesis of Pelvic Endometriosis. Hum. Reprod..

[20] Proestling K., Birner P., Gamperl S., Nirtl N., Marton E., Yerlikaya G., Wenzl R., Streubel B., Husslein H. (2015). Enhanced Epithelial to Mesenchymal Transition (EMT) and Upregulated MYC in Ectopic Lesions Contribute Independently to Endometriosis. Reprod. Biol. Endocrin..

[21] Zheng Q., Xu Y., Lu J., Zhao J., Wei X., Liu P. (2016). Emodin Inhibits Migration and Invasion of Human Endometrial Stromal Cells by Facilitating the Mesenchymal-Epithelial Transition through Targeting ILK. Reprod. Sci..

[22] Hawi Z., Tong J., Dark C., Yates H., Johnson B., Bellgrove M.A. (2018). The Role of Cadherin Genes in Five Major Psychiatric Disorders: A Literature Update. Am. J. Med. Genet. B.

[23] Devaux C.A., Mezouar S., Mege J.L. (2019). The E-Cadherin Cleavage Associated to Pathogenic Bacteria Infections Can Favor Bacterial Invasion and Transmigration, Dysregulation of the Immune Response and Cancer Induction in Humans. Front. Microbiol..

[24] Guidi A., Laricchia-Robbio L., Gianfaldoni D., Revoltella R., Del Bono G. (2001). Comparison of a Conventional Immunoassay (ELISA) with a Surface Plasmon Resonance-Based Biosensor for IGF-1 Detection in Cows’ Milk. Biosens. Bioelectron..

[25] Ulman A. (1996). Formation and Structure of Self-Assembled Monolayers. Chem. Rev..

[26] Schlenoff J.B., Li M., Ly H. (1995). Stability and Self-Exchange in Alkanethiol Monolayers. J. Am. Chem. Soc..

[27] Schreiber F. (2004). Self-Assembled Monolayers: From “Simple” Model Systems to Biofunctionalized Interfaces. J. Condens. Matter Phys..

[28] Schwartz D.K. (2003). Mechanisms and Kinetics of Self-Assembled Monolayer Formation. Annu. Rev. Phys. Chem..

[29] Kastl K., Ross M., Gerke V., Steinem C. (2002). Kinetics and Thermodynamics of Annexin A1 Binding to Solid-Supported Membranes: A QCM Study. Biochemistry.

[30] FDA Bioanalytical Method Validation Guidance for Industry Bioanalytical Method Validation 2018, 1–44. https://www.fda.gov/regulatory-information/search-fda-guidance-documents/bioanalytical-method-validation-guidance-industry.

[31] (2022). Committee for Medicinal Products for Human Use ICH M10 on Bioanalytical Method Validation—Scientific Guideline European Medicines Agency. https://www.ema.europa.eu/en/ich-m10-bioanalytical-method-validation-scientific-guideline.

[32] Hamerlik P., Lathia J.D., Rasmussen R., Wu Q., Bartkova J., Lee M.H., Moudry P., Bartek J., Fischer W., Lukas J. (2012). Autocrine VEGF-VEGFR2-Neuropilin-1 Signaling Promotes Glioma Stem-like Cell Viability and Tumor Growth. J. Exp. Med..

[33] Sharada P., Swaminathan U., Nagamalini B.R., Kumar K.V., Ashwini B.K., Lavanya V.L.N. (2018). Coalition of E-Cadherin and Vascular Endothelial Growth Factor Expression in Predicting Malignant Transformation in Common Oral Potentially Malignant Disorders. J. Oral. Maxillofac. Pathol..

[34] Leach L., Gray C., Staton S., Babawale M.O., Gruchy A., Foster C., Mayhew T.M., James D.K. (2004). Vascular Endothelial Cadherin and β-Catenin in Human Fetoplacental Vessels of Pregnancies Complicated by Type 1 Diabetes: Associations with Angiogenesis and Perturbed Barrier Function. Diabetologia.

[35] Di Raimondo F., Azzaro M.P., Palumbo G.A., Bagnato S., Stagno F., Giustolisi G.M., Cacciola E., Sortino G., Guglielmo P., Giustolisi R. (2001). Elevated Vascular Endothelial Growth Factor (VEGF) Serum Levels in Idiopathic Myelofibrosis. Leukemia.

[36] Naik A., Al-Zeheimi N., Bakheit C.S., Al Riyami M., Al Jarrah A., Al Moundhri M.S., Al Habsi Z., Basheer M., Adham S.A. (2017). Neuropilin-1 Associated Molecules in the Blood Distinguish Poor Prognosis Breast Cancer: A Cross-Sectional Study. Sci. Rep..

[37] Otoo J.A., Schlappi T.S. (2022). REASSURED Multiplex Diagnostics: A Critical Review and Forecast. Biosensors.

[38] Land K.J., Boeras D.I., Chen X.S., Ramsay A.R., Peeling R.W. (2018). REASSURED Diagnostics to Inform Disease Control Strategies, Strengthen Health Systems and Improve Patient Outcomes. Nat. Microbiol..

[39] Miao P., Tang Y. (2020). DNA Walking and Rolling Nanomachine for Electrochemical Detection of MiRNA. Small.

